# Alarm fatigue mitigation through nurse empowerment: a pre-post intervention study in two intensive care units

**DOI:** 10.1186/s12912-025-03613-9

**Published:** 2025-08-05

**Authors:** Reut Ron, Itzik Barnett, Ruti Berger, Sarah Sberro-Cohen

**Affiliations:** 1 Assuta Health Services Research Institute, Tel-Aviv, Israel; 2 Ben-Gurion University of the Negev, Beer-Sheva, Israel; 3 General Intensive Care Unit, Assuta Ashdod University Hospital, Ashdod, Israel; 4 Maccabi Healthcare Services, Southern District, Omer, Israel

**Keywords:** Alarm fatigue, Intensive care unit, Authority delegation, Nursing staff, Patient safety, Nurse empowerment

## Abstract

**Background:**

Alarm fatigue in intensive care units (ICUs) is a pressing issue that jeopardizes patient safety and staff well-being. In Israel, although hospitals are permitted to determine who sets alarm thresholds, most have historically assigned this authority exclusively to physicians. This stems from the absence of national policy and institutional reluctance, driven by risk management and physician resistance to transfer clinical responsibilities to nurses, limiting timely responses to patient needs.

**Methods:**

This was a prospective pre-post intervention study using multiple data sources, including structured observations and staff surveys, conducted in pediatric and adult general ICUs at Assuta Ashdod University Hospital. The intervention involved transferring alarm threshold-setting authority for bedside monitor alarms from physicians to nursing staff, supported by a comprehensive training program. Evaluation included structured observations of alarm events (435 pre- and 288 post-intervention), staff surveys (*n* = 33 pre, *n* = 24 post), and feedback on the implementation process.

**Results:**

The primary outcome, alarm response rate—defined as the proportion of monitor alarms that elicited any observable staff reaction—increased slightly in the pediatric ICU, from 65 to 69%, and remained unchanged (50%) in the adult ICU. Notably, 90% of nurses in the pediatric ICU and 75% in the adult ICU reported increased confidence in setting alarm thresholds independently. Additionally, nursing staff expressed greater awareness of alarm fatigue and improved trust in alarm systems.

**Conclusions:**

Empowering nursing staff by delegating alarm threshold authority resulted in enhanced alarm management and increased self-efficacy among nurses, though outcomes varied between units. These findings highlight the need for national guidelines to support this delegation while considering the unique characteristics of each ICU.

**Trial registration:**

Not applicable.

**Supplementary Information:**

The online version contains supplementary material available at 10.1186/s12912-025-03613-9.

## Background

Alarm fatigue, as defined by the American Association of Critical Care Nurses (AACN), is an auditory sensory overload that occurs when healthcare staff are exposed to an excessive level of alarms, leading to desensitization to alarm sounds, decreased auditory awareness, and minimal responses to alerts, ultimately compromising patient safety [1]. In intensive care units (ICUs), the prevalence of frequent and often nonactionable alarms, also known as false alarms, which are alerts triggered by technical or physiological deviations that do not require clinical intervention, creates an environment of chronic overstimulation and alarm desensitization [2, 3]. The extent of the problem is well documented. Weekly alarm count in ICUs can reach one million, with up to 85% considered false alarms. A children’s hospital reported 5,300 alarms per day, 95% of which were false [4]. Studies have shown that less than 15% of alarms are clinically relevant or actionable [5]. The primary cause of false alarms is setting threshold limits that do not align with the patient’s current medical condition [6]. Alarms are generated by a wide range of devices—including monitors, pumps, ventilators, incubators, and call systems, contributing to an overwhelming acoustic environment [5, 7, 8]. One of the main sources of alarms is the patient monitor—a device that continuously tracks multiple physiological parameters such as heart rate, blood pressure, and oxygen saturation.

Alarm fatigue poses serious risks for both patients and staff. From a patient safety perspective, desensitization to alarms can lead to missed critical events and adverse outcomes, including fatalities [3, 9]. The U.S. Food and Drug Administration (FDA) has attributed over 500 alarm-related patient deaths over five years, with alarm fatigue identified as a contributing factor [9]. From a workforce perspective, alarm fatigue is associated with cognitive overload, stress, and burnout. Staff report confusion in decision-making due to simultaneous alarms, impaired professional performance, and emotional exhaustion [2, 3, 10]. In response to these risks, the Joint Commission International (JCI) undertook an initiative to reduce alarm fatigue in the beginning of 2013. The organization called for hospitals to reduce alarm fatigue by improving alarm relevance, clarifying authority, and implementing training and protocols [11]. Low response rates to alarms pose a critical threat to patient safety, as unattended or delayed responses can result in missed clinical deterioration. This challenge is exacerbated by alarm fatigue and desensitization, contributing to preventable harm.

Within the Israeli context, and the absence of national policy, hospitals have independently defined alarm management protocols. From a risk management standpoint, many institutions have chosen to retain alarm-setting authority solely with physicians, viewing this as a safer and more legally defensible approach. Moreover, physician resistance to transferring clinical authority to nurses has further limited changes in practice. Consequently, nurses are limited to narrow adjustment ranges and often cannot tailor settings to patient needs, despite spending the most time at the bedside. An earlier Israeli study investigated the gap between policy and practice concerning nurses’ authority in ICUs. Despite policies assigning certain responsibilities to nurses, significant disparity remains in their actual autonomy. This underscores the importance of examining organizational dynamics and addressing systemic barriers to collaborative practice and care improvement [12]. Empirical studies support interventions such as personalized alarm thresholds [13–15], educational programs, protocols and clinical workflow optimization [3, 16–20] as strategies to reduce false alarms and improve nurse responsiveness. Since nursing staff spend most of their time in the ICU alongside patients, knowing their needs and habits, responding to alarms [21], delegating authority to nursing staff has numerous positive impacts, including opportunities for leadership skill development, empowerment, and using these skills to improve management capabilities [22].

A 2013 Israeli report from a neonatal intensive care unit [23] examining professional boundaries between nursing and medical staff in special care units for newborns (NICUs), reported that nurses demonstrated a sense of capability and high motivation to expand decision-making authorities. Physicians showed willingness to delegate certain operational areas to nursing authority, helping to alleviate the staffing shortage, as this delegation addressed the limited availability of physicians.

Nurse burnout is a critical challenge [24]. Empowering nurses is associated with reduced burnout and lower turnover intentions [25]. Strategies include involving nurses in decision-making, increasing autonomy and supporting goal achievement [26]. In this context, delegating alarm threshold-setting authority to nursing staff not only addresses alarm fatigue but also fosters empowerment, contributing to burnout prevention.

Despite increasing international recognition of these strategies, few studies have addressed alarm fatigue and nurse empowerment in the Israeli context. The lack of standardized national guidelines results in significant variability in alarm management practices across hospitals, leaving individual units to develop their own protocols.

This study aimed to address this gap by evaluating the impact of delegating alarm threshold-setting authority from physicians to ICU nurses. Using a mixed-methods approach, we assessed changes in alarm response behaviors, staff perceptions of alarm fatigue, and implementation feasibility. The study focused on behavioral and perceptual outcomes, rather than alarm reduction per se, to inform future policy and support safer, more collaborative practices in intensive care settings.

## Methods

### Study design, setting and participants

This was a prospective pre-post intervention study with a multi-source evaluation approach. Data sources—including structured observations, staff surveys, and implementation feedback—were used to evaluate both behavioral outcomes and staff perceptions. This multi-source approach enabled a more comprehensive understanding of the intervention’s feasibility and staff experience within the ICU setting, in line with guidance on process evaluations of complex interventions that emphasize capturing both implementation processes and contextual factors [27].

The study was conducted in two ICUs within a public hospital in Israel: the pediatric ICU (PICU) and the adult general ICU (GICU). Data were collected through structured observations, staff surveys, and feedback questionnaires.

The study included all permanent clinical staff (*n* = 62) from the PICU and GICU, comprising 40 nurses, eight attending physicians, and 14 medical residents. Inclusion criteria required staff to have at least six months of ICU experience. Staff on long-term leave during the intervention period were excluded. Participation in the survey components was open to all eligible staff members, with pre- and post-intervention questionnaires distributed electronically. Response rates were 53% (*n* = 33) and 39% (*n* = 24), respectively. Feedback questionnaires were completed by nurses who had completed the digital training module, representing a convenience sample of engaged participants.

Observational data were collected independently of individual staff participation, based on a predefined shift allocation schedule to ensure representation across units and time periods. Observers recorded alarm events at occupied beds with unobstructed visibility, without targeting specific individuals.

### The intervention - training program

The intervention involved delegating the authority to set monitor alarm thresholds from physicians to nursing staff, following a tailored training program. Each department developed new protocols and treatment guidelines, including structured definitions of normal vital sign ranges by age in PICU and by patient condition in GICU. These materials, along with background information and practical examples, were consolidated into a staff training module.

The training module included scientific and adult general background on alarm fatigue and mitigation strategies, demonstration videos, unit-specific parameter setting guidelines, and case-based assessment questions. Validation of the training content was conducted by ICU experts, and adjustments were made to meet the specific needs of each unit. The module was constructed in a format compatible with the hospital’s digital education system and was launched in October 2023 for all nursing staff in both departments.

Face-to-face training sessions were conducted during staff meetings and individually during initial shifts following module completion. After completing the training module and receiving approval from department head nurses, nursing staff began independently setting monitor alarm parameters, consulting with and obtaining physician approval for complex and unstable cases when necessary.

Following module completion and department approval, nurses began independently setting alarm thresholds. In complex or unstable cases, they were advised to consult physicians. No formal process was established for routine verification of alarm settings by senior staff at the start of each shift.

### Data collection procedures – structured observations

A two-phase observational study was conducted both before and after the intervention. Trained observer pairs, who underwent specific training in standardized data collection and alarm classification, conducted 30-minute observations. Observers were randomly assigned to shifts using a predefined allocation schedule to ensure representative sampling across morning, evening, and night shifts. To minimize the Hawthorne effect, staff were informed only that the study examined GICU activities, with the specific focus on alarm fatigue remaining undisclosed. Observers were strategically positioned at the nurses’ station and at a secondary location offering optimal unit visibility (i.e., a clear, unobstructed line of sight to the patient’s bedside monitor and staff activity). They monitored alarm events from patient monitoring equipment at designated observation points.

The observation protocol and documentation instruments were developed through comprehensive literature review of similar studies [28–30]. These tools underwent rigorous content validation through expert panel review and pilot testing. During the pilot phase, real-time observations were conducted in the ICU by observer pairs using the full documentation protocol. Each observer independently recorded alarm events and staff responses on separate forms, and comparisons between the forms revealed no missed alarms. This validation confirmed the feasibility of real-time latency tracking. Accordingly, all study observations were conducted by trained observer pairs, with one observer focused on timing response latency and the other maintaining continuous surveillance for new alarms.

Observations were conducted by pairs of nursing students completing their clinical training at the hospital. The observers were not members of the ICU staff and received prior training in standardized alarm classification and data collection procedures.

Staff response was defined as any observable behavioral reaction by clinical personnel following the initiation of an alarm. Based on established frameworks from prior studies, responses were categorized by the observers into three main types: a brief visual orientation toward the source of the alarm without physical movement toward the patient (referred to as head-raising or visual check), remote handling of the alarm from the nurses’ station or central monitoring system (such as silencing or resetting), and direct bedside intervention, where staff entered the patient’s room and interacted with the patient or equipment. These classifications were determined in advance and applied consistently by trained observers.

Data record included alarm origin, severity (yellow or red, based on the hospital monitor classification system), staff response type (visual check, remote management, bedside intervention), and response latency (seconds from alarm initiation). Only occupied beds with unobstructed visibility were included in the observation protocol. Patients receiving active care or with privacy curtains drawn were excluded from observation periods.

During the structured observation sessions, all audible clinical alarms from bedside equipment were recorded, including those from patient monitors, ventilators, and infusion pumps, in order to capture the full acoustic environment experienced by staff during ICU shifts. Observers documented the source device, severity level (e.g., red, yellow), and contextual factors such as staff presence at the bedside, patient mobilization, or equipment disconnection. This allowed for later distinction between clinically relevant and irrelevant alarms.

However, since the intervention specifically targeted alarm threshold settings on bedside monitors—the most frequent source of alarms and the only device type for which nursing staff were granted authority to modify parameters—only monitor-generated alarms were included in the primary outcome analysis. Moreover, only those alarms that were audible, originated from a monitor, and were eligible for response based on predefined criteria (e.g., staff not already present) were analyzed for alarm response behavior. Alarms judged irrelevant or non-actionable were excluded. This filtering approach ensured consistency and validity in assessing the effects of the intervention on staff alarm responsiveness.

### Data collection procedures – staff surveys and feedback questionnaires

As no single validated instrument existed that fully addressed our research goals in the ICU context, we developed study-specific pre- and post-intervention questionnaires. These tools were designed to assess alarm fatigue prevalence, staff perceptions, and attitudes toward the new policy. Items were based on a comprehensive review of the literature and adapted from validated questionnaires [19, 31, 32], then reviewed by an expert panel and pilot-tested for clarity and consistency.

The pre- and post-intervention questionnaires each included thirty-four items, primarily closed-ended Likert-scale questions (ranging from “strongly disagree” to “strongly agree”), with several open-ended prompts. Items addressed key domains such as exposure to alarm burden, behavioral response patterns, trust and desensitization, perceived urgency and emotional impact (e.g., anxiety, confusion), confidence in alarm threshold settings, device-specific responses (e.g., monitor, ventilator), and institutional factors such as the use of alarm protocols. The same instrument was used at both timepoints to ensure comparability. The questionnaire underwent expert panel content validation and pilot testing for clarity and internal consistency.

In this study, alarm fatigue was operationally defined as a multidimensional phenomenon encompassing perceived alarm burden, emotional desensitization, cognitive overload, reduced responsiveness, and diminished trust in alarms. These dimensions were assessed through survey items covering staff perceptions, emotional reactions, response tendencies, and attitudes toward alarm relevance and frequency.

As survey responses were anonymous and not linked across timepoints, the pre- and post-intervention datasets reflect independent samples. Individual-level changes could not be assessed, and between-group comparisons were used to evaluate changes over time.

An interim implementation and usability survey was distributed in paper format two weeks after the training module launch, targeting nurses and physicians who had completed the digital training. This 14-item questionnaire included eleven items for nurses and three for physicians. Nurse items evaluated training clarity and usability, confidence in setting alarm thresholds, perceived changes in attentiveness, trust, patience, and continued reliance on physician consultation. One item specifically assessed whether alarm threshold-setting remained complex. Physician items addressed trust in nurses’ threshold-setting abilities, preferences regarding consultation, and perceived changes in nurses’ responsiveness.

Participation in all surveys was voluntary and anonymous. The pre- and post-intervention questionnaires were distributed via digital links in staff WhatsApp groups, while the interim survey was collected in person by unit coordinators.

Data collection occurred in three waves: baseline (July–September 2022), interim (January 2024, two weeks post-module release), and follow-up (March 2024, two months post-intervention). This structured timeline enabled a comprehensive evaluation of the intervention’s impact while maintaining methodological rigor.

### Sample size and statistical analysis

The primary outcome of the study was the alarm response rate, defined as the proportion of alarms that elicited any observable response from nursing staff during the 30-minute observation sessions. A response was considered valid if it involved a visual check, remote management, or bedside intervention, as documented by trained observers. An alarm was defined as an audible alert generated by the bedside patient monitor, classified either as red (high severity) or yellow (moderate severity) according to the hospital’s monitor system. Alarms from other devices (e.g., ventilators, infusion pumps) were recorded during observations but were not included in the primary outcome analysis, as the intervention targeted only monitor-based threshold settings.

The primary outcome measure, “alarm response rates,” guided sample size calculations using Select Statistical Services’ statistical power calculator. Parameters included significance level of 95% (α = 0.05), statistical power of 80% (β = 0.20), baseline response rate of 46.9% derived from systematic literature review, and anticipated improvement of 10% increase in appropriate response rates. These parameters yielded a required sample size of 224 alarm events per study phase.

All statistical analyses were conducted using R (version 4.4.0), a free and open-source statistical computing environment maintained by the R Core Team (2025). Descriptive statistics—including absolute frequencies and relative percentages—were calculated for categorical variables such as alarm response types (e.g., visual orientation, remote intervention, bedside entry, no response).

Alarm response patterns were analyzed across intervention periods (pre vs. post) and units (Pediatric ICU and General ICU), stratified by device type. Chi-squared tests of independence were used to evaluate differences in response type distributions across time points within each unit-device stratum. Fisher’s exact test was applied when expected cell counts were below 5.

Overall response rates (i.e., the proportion of alarms with any observable staff response) were compared using 2 × 2 contingency tables and tested for significance using either chi-squared or Fisher’s exact tests, as appropriate. All analyses used a two-tailed significance threshold of *p* < 0.05.

### Ethics approval and consent to participate

The study was approved by the Ethics Committee of Assuta Ashdod Hospital (IRB reference number AAA-0102-20), ensuring compliance with institutional guidelines and national regulations. All procedures involving human participants were conducted in accordance with the ethical standards of the institutional and national research committee and with the 1964 Declaration of Helsinki and its later amendments. As the study involved hospital staff only (and no patients), and data were collected through structured workplace observations and anonymous surveys without any personally identifiable information, the Ethics Committee granted a waiver of informed consent.

This manuscript adheres to the StaRI (Standards for Reporting Implementation Studies) checklist [33], which provides guidance for transparent reporting of implementation strategies and their evaluation within healthcare settings. The completed StaRI checklist is submitted as a supplementary file.

Funding was provided through a research grant awarded by the Israel National Institute for Health Policy Study (NIHP) number 2020/227. The funding organization had no involvement in the study design, data collection, analysis, interpretation, or manuscript preparation.

## Results

The results presented below are derived from three data sources: structured observations of alarm response behavior, pre- and post-intervention staff surveys, and an interim implementation and usability questionnaire. Statistical significance was assessed using paired t-tests for pre-post comparisons within units, ANOVA for between-unit differences, and chi-square tests for categorical variables such as response types.

### Observations based assessment of alarm fatigue

Observations were conducted during July–September 2022 for the pre-intervention phase and during March 2024 for the post-intervention phase. A total of 723 audible alarm events were observed across 43 structured observation sessions, each lasting 30 min. While alarms from various bedside equipment (including ventilators and infusion pumps) were heard and noted during observations, only alarms generated by the bedside patient monitors were included in the outcome analysis, as these were the exclusive focus of the intervention.

In the pre-intervention phase, 23 shifts were observed, 10 in the PICU and 13 in the GICU. In the post-intervention phase, 20 shifts were observed, 8 in the PICU and 12 in the GICU. Observations were scheduled to ensure representation across morning, evening, and night shifts. Observers documented alarm events without targeting specific patients or staff members. Each observation session included only occupied beds with unobstructed visibility. In the PICU, the number of beds observed per session ranged from 1 to 4 (mean: 2.6 pre, 3.1 post). In the GICU, 3 to 5 beds were typically observed per session (mean: 3.6 pre, 4.1 post), ensuring consistency across study phases.

Of the 723 alarms, 435 recorded in the pre-intervention phase (PICU: 130, GICU: 305) and 288 in the post-intervention phase (PICU: 160, GICU: 128). Across both units, the majority of alarms were categorized as yellow, indicating moderate severity. In the post-intervention period, as in the pre-intervention period, monitoring devices remained the primary source of alarms, with a notable increase in the proportion of yellow alerts compared to red alerts (Table 1).

**Table 1:** Observational data summary pre and post the intervention, by ICU unit

		General ICU		Pediatric ICU	
		Pre	Post	Pre	Post
Shifts (N)		13	12	10	8
Alarms (N)		305	128	130	160
Alarms with no bedside staff (N)		204	90	89	138
Beds under observation	Sum	739	365	237	416
	Average	3.62	4.06	2.66	3.01
	Range	3–4	3–5	1–3	2–4
Number of Patients in the unit	Sum	1606	776	364	481
	Average	7.87	8.62	4.09	3.49
	Range	5–10	7–10	2–6	2–5
Number of Nurses in the unit	Sum	948	450	218	353
	Average	4.65	5.00	2.45	2.56
	Range	3–5	5–5	2–3	2–3
Alarm severity	Red	22%	24%	11%	12%
	Yellow	66%	72%	47%	79%

ICU = Intensive Care Unit

Pre = Before the intervention (July–September 2022); Post = After the intervention (March 2024)

Alarms with no staff at bedside refer to events where the alarm was triggered in a bed without nursing staff present at the time of onset

**Fig. 1:**
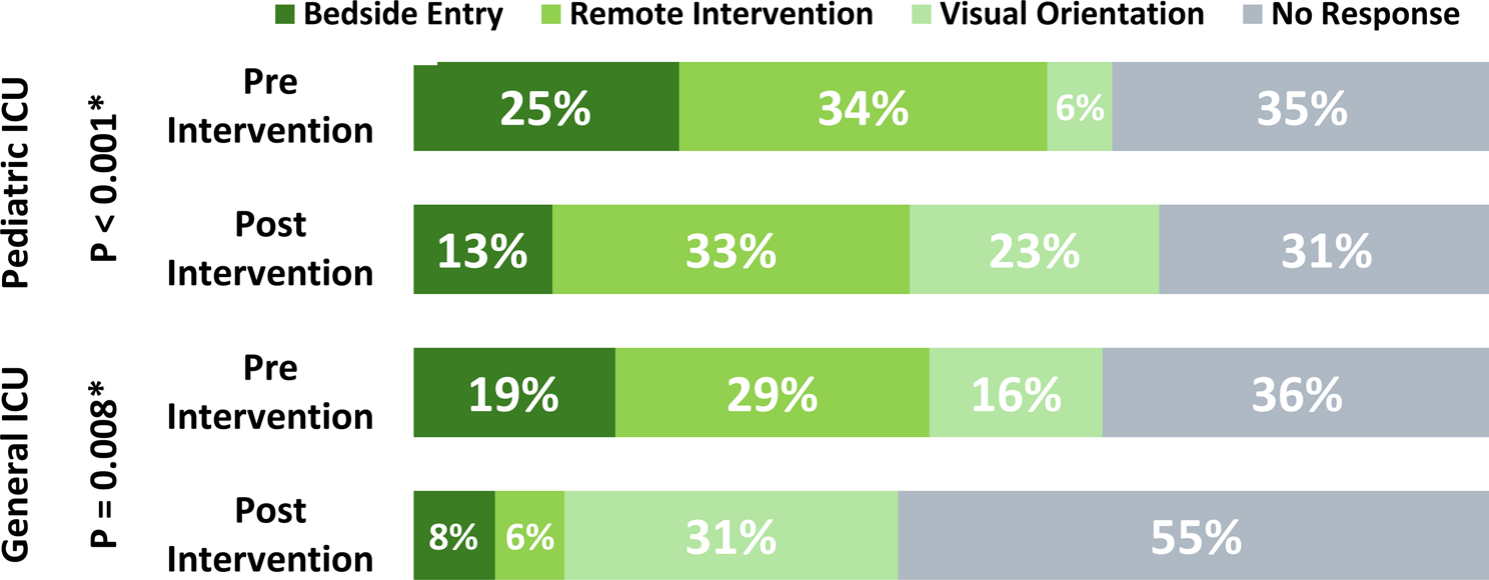
Observation results-staff response by unit and intervantion period. Bedside entry – the staff member physically entered the patient’s room in response to the alarm. Remote Intervention – Action was taken from the nurse’s station or control panel without entering the room. Visual Orientation – The staff responded by visually checking the monitor or patient from outside the room. No Response – No observable reaction occurred within the observation window. Note: Percentages reflect the distribution of first observed staff responses to monitor-based alarms during structured observation sessions. “Pre Intervention” = baseline period (July–September 2022); “Post Intervention” = follow-up period (March 2024). *p-values* (left axis) are based on Chi-squared tests comparing pre- and post-intervention response type distributions within each ICU unit

In the PICU, the proportion of addressed alarms increased modestly from 65% pre-intervention to 69% post-intervention; however, this change was not statistically significant (χ² = 0.23, *p* = 0.63; Table 2). In contrast, response rates to ventilator alarms slightly decreased from 64 to 60%, also without statistical significance (*p* = 1.00). While overall response rates remained relatively stable, a significant shift was observed in the distribution of response types (*p* < 0.001; Fig. 1). The proportion of responses involving visual orientation (e.g., head-raising without room entry) increased from 6 to 23%, and the rate of bedside entry declined from 25 to 13%, suggesting an increased reliance on remote or non-intrusive monitoring strategies.

**Table 2:** Pre- and post-intervention alarm response rates by unit (PICU vs. GICU) and device type (Monitor vs. Ventilator)

Unit	Device	Response rate		Test statistic	p-value	Test type
		Pre	Post			
Pediatric ICU	Monitor	64.9% (*n* = 77)	69.2% (*n* = 133)	0.23	0.632	Chi-squared
	Ventilator	63.6% (*n* = 11)	60.0% (*n* = 5)		1	Fisher’s Exact
General ICU	Monitor	64.1% (*n* = 117)	44.9% (*n* = 78)	6.278	0.012*	Chi-squared
	Ventilator	32.2% (*n* = 87)	87.5% (*n* = 8)		0.003*	Fisher’s Exact

Response rate reflects the percentage of alarms that received staff attention

“Pre” = before intervention; “Post” = after intervention. Sample sizes (n) refer to the number of monitor-based alarm observations analyzed. *p < 0.05 indicates statistical significance

In the GICU, the proportion of addressed alarms decreased significantly from 64% pre-intervention to 45% post-intervention (χ² = 6.28, *p* = 0.012; Table 2). For ventilator alarms, a sharp increase in response rate was observed—from 32 to 87% (*p* = 0.003; Table 2). Regarding monitor alarms specifically, the distribution of response types also shifted notably post-intervention (*p* < 0.001; Fig. 1). While visual orientation responses rose substantially from 6 to 23%, bedside entry responses declined from 25 to 13%, suggesting a transition toward less intrusive monitoring approaches.

When comparing alarm responses by device type, different trends emerged. For monitor alarms, the percentage of addressed alarms decreased from 64% before the intervention to 36% after the intervention. This decrease was not statistically significant (*p* = 0.44) but highlights a shift in attention.

As a secondary analysis, response time (in seconds) was examined after excluding extreme outliers above 20 s. In the PICU, the number of valid observations was 57 in the pre-intervention phase and 94 post-intervention. The mean response time increased slightly from 1.82 s (SD = 3.19, median = 0, range = 0–12) to 2.33 s (SD = 3.74, median = 1.5, range = 0–18). This change was not statistically significant (*t*-test *p* = 0.38; Wilcoxon *p* = 0.16).

In the GICU, 101 valid observations were recorded pre-intervention and 38 post-intervention. Here, the mean response time increased from 3.17 s (SD = 4.33, median = 2, range = 0–20) to 4.68 s (SD = 4.47, median = 3, range = 0–20). While the increase was not significant in the t-test (*p* = 0.08), the Wilcoxon test yielded a marginally significant difference (*p* = 0.011).

### Survey based assessment of alarm fatigue

A total of 62 clinical staff members were eligible to participate in the study, including 40 nurses, 8 attending physicians, and 14 medical residents across two ICUs. The pre- and post-intervention surveys, as well as the interim feedback questionnaire, were completed by a combined total of 71 responses. In the Pediatric ICU (PICU), 12 staff members responded to the baseline survey (10 nurses, 2 physicians), 14 responded to the interim survey (12 nurses, 2 physicians), and 10 completed the post-intervention survey (all nurses). In the adult General ICU (GICU), 21 staff members responded at baseline (20 nurses, 1 physician), and 14 responded post-intervention (12 nurses, 2 physicians). No interim surveys were completed in the GICU.

Demographic characteristics of survey respondents reflected the ICU workforce composition. Of the 71 participants, 64 were nurses and 7 were physicians. The majority identified as female (55 out of 71), with a mean age of 41 years (range: 27–64). Most respondents held academic nursing degrees, including 35 with a bachelor’s degree and 19 with a master’s degree. Respondents were nearly evenly distributed between the two units, with 36 from the PICU and 35 from the GICU.

All nurses who completed the training module were invited to complete the interim survey. Observational data were collected independently of survey participation and without tracking specific individuals.

The survey-based assessment of alarm fatigue revealed complex patterns of staff perception and response across two ICUs. Survey participation rates were 53% pre-intervention (33/62 respondents) and 39% post-intervention (24/62 respondents), providing insights into staff experiences with medical alarms.

Alarm desensitization showed notable increases in both units, with the PICU demonstrating a rise from 17 to 30% and the GICU experiencing a more pronounced increase from 29 to 43%. Staff awareness of alarm-related challenges significantly expanded, particularly evident in their responses to simultaneous alarms and professional interference. In the PICU, the agreement that multiple alarms cause confusion dramatically increased from 25 to 70%, while in the GICU, this increased from 24 to 36%. Similarly, the acknowledgment of alarm sounds preventing optimal professional performance rose substantially in the PICU from 33 to 70%, with the GICU showing a comparable increase from 24 to 36%.

The practical manifestation of alarm fatigue, measured by self-report of not hearing alarms, demonstrated significant variability. The PICU showed an increase from 17 to 30%, while the GICU exhibited a more dramatic rise from 5 to 21%. Staff responsiveness perceptions diverged between units, with the PICU improving from 42 to 70% and the GICU declining from 71 to 57%. These findings merit careful interpretation, as they may reflect both actual increases in alarm fatigue and an enhanced awareness of the phenomenon.

Interestingly, alarm tolerance metrics revealed unexpected improvements. Agreement that repeated alarms lead to lost patience decreased in both units (PICU: 58–40%; GICU: 52–36%). The PICU completely eliminated reported indifference to recurring alarms (25–0%), while the GICU showed an increase from 19 to 43%. For more details, please refer to Table 3.

**Table 3:** Proportion of ICU staff agreeing with alarm-related statements before and after the intervention, by unit type

	Adult ICU		Pediatric ICU	
	Before (*N* = 21)	After (*N* = 14)	Before (*N* = 12)	After (*N* = 10)
Over time, my sensitivity to alarms decreases	29%	43%	17%	30%
Multiple alarms sound simultaneously confuse me when I need to make decisions	24%	36%	25%	70%
Alarm sounds prevent me from performing my professional duties optimally	24%	36%	33%	70%
Sometimes I don’t hear the alarm at all	5%	21%	17%	30%
The staff is sensitive to alarms and responds quickly	71%	57%	42%	70%
When alarms repeat over and over, I lose my patience	52%	36%	58%	40%
When alarms are repetitive, I become indifferent to them	14%	43%	25%	0%
I wait before responding to an alarm sound, hoping it will stop on its own	19%	43%	17%	0%
Following the training and my responsibility for setting alarm limits: I am more attentive to alarms and alerts		67%		100%
I have greater confidence in the alarms and alert		67%		83%
I am more patient with the alarms and alerts		58%		83%

Note. Responses reflect the percentage of participants who agreed or strongly agreed with each statement on a 5-point Likert scale. The intervention included staff training and delegation of alarm parameter settings to nurses. “Adult ICU” refers to the General Intensive Care Unit (GICU); “Pediatric ICU” refers to the Pediatric Intensive Care Unit (PICU)

Post-intervention outcomes highlighted the impact of increased staff autonomy in alarm management. In the PICU, 100% of respondents reported increased alarm attentiveness, and 83% indicated enhanced trust and patience. The GICU demonstrated more moderate improvements, with 67% reporting increased attentiveness and trust, and 58% showing improved patience towards alarms.

### Team feedback on the learning module and intervention

During the two weeks post-intervention, 92% of GICU staff and 100% of PICU staff completed the digital training module. Most respondents (90% in PICU, 75% in GICU) found the training clear and informative. Despite this, 33% of GICU staff reported that setting alarm thresholds remained a complex process compared to none in the PICU. In both units, most respondents reported that they independently set alarm limits in the previous week and felt confident in doing so (90% of in the PICU and 75% in the GICU). Nevertheless, most nurses still reported consulting with physicians on this matter (50% PICU and 75% GICU). 100% of respondents in the PICU and 67% in the GICU reported high satisfaction with the ability to independently set alarm limits.

Practical implementation of the intervention was assessed through staff agreement with several behavioral statements, including the reverse-coded item: *“At the beginning of my shift*,* I set alarm limits to a wider range than desired.”* Lower agreement with this statement was interpreted as a positive indicator of effective implementation, reflecting greater adherence to clinically appropriate threshold settings.

These findings are based on responses to the interim implementation and usability survey, which was administered two weeks after the training module launch to a convenience sample of nurses and physicians who had completed the module. The interim survey focused on perceived confidence, behavioral change, and alignment with the new protocol.

In the PICU, implementation outcomes were particularly strong: prior to the intervention, only 10% of nurses reported regularly setting alarm limits independently (frequently or always), whereas post-intervention, 50% reported doing so regularly, and an additional 20% reported occasional implementation. In the GICU, 17% of nurses reported regularly applying the new practice post-intervention, compared to 10% prior, with an additional 33% reporting occasional implementation. These findings are detailed in Table 4.

**Table 4:** Percentage of ICU staff indicating agreement with statements about the training module and post-intervention practice, by unit type

	Adult ICU (*N* = 14)	Pediatric ICU (*N* = 14)
I completed the training module on setting alert thresholds	92%	100%
The training module was clear/understandable to me	92%	90%
The training module was easy to complete	83%	100%
The training module effectively conveyed information about setting alert thresholds	75%	90%
Despite the training module, I still feel that setting alert thresholds is a complicated process	33%	0%
In the past week, I have personally set alert thresholds	75%	90%
I feel confident setting alert thresholds for my patients on my own	75%	100%
I still consult with a physician regarding setting alert thresholds	75%	50%
I am satisfied with the ability to set alert thresholds on my own	67%	100%

Note. Percentages reflect staff members who selected “Agree” or “Strongly Agree” on a 5-point Likert scale. Both groups (General ICU and Pediatric ICU) completed the training module before the final implementation of the delegation protocol for alarm parameter adjustments

### Nursing and medical staff attitudes regarding monitor alarm setting authority

Questions regarding the practical implementation of limit setting and staff attitudes on this matter were included in both the first and third questionnaires. Data analysis was conducted separately for nursing and medical staff. Regarding the statement “Monitor alarm limit decision-making authority can be extended to nursing staff (in addition to physicians),” in the PICU, 90% of nursing staff responded “frequently” or “almost always” prior to the intervention, with a decrease to 80% post-intervention, while 20% indicated “sometimes.” The GICU showed an opposite trend, with 70% of nursing staff responding “frequently” or “almost always” pre-intervention, increasing to 83% post-intervention. Concerning medical staff (from both departments combined), dissatisfaction or lack of confidence emerged following the intervention, with 67% selecting “frequently” or “almost always” pre-intervention, compared to 50% indicating “sometimes” and 50% “almost never” post-intervention.

Similarly, staff responses to the statement “My institution effectively utilizes clinical policies and procedures for alarm management” showed notable patterns. In the PICU, 80% of nursing staff responded “sometimes” pre-intervention, while post-intervention, 50% responded “frequently” or “almost always.” GICU demonstrated an opposite trend, with 60% of nursing staff responding “frequently” or “almost always” pre-intervention, decreasing to 50% post-intervention. Regarding medical staff (from both departments combined), dissatisfaction or lack of confidence was observed following the intervention, with pre-intervention responses showing 67% selecting “sometimes” and 33% “frequently,” compared to post-intervention responses of 50% indicating “sometimes” and 50% “almost never.”

All responding physicians strongly or very strongly agreed with statements regarding their trust in properly trained nurses to appropriately set patient alarm limits, and that nurses are more attentive and patient with alarms now that they set the alarm limits themselves. However, all physicians also agreed with the statement “I prefer that nurses still consult with me before changing alarm limits”.

## Discussion

This prospective pre-post intervention study, examined the impact of delegating alarm threshold-setting authority from physicians to nursing staff in two intensive care units (PICU and GICU). The study yielded nuanced insights into the impact of such interventions on alarm fatigue, staff attitudes, and clinical practices.

Findings were drawn from three complementary sources: structured alarm observations, pre/post staff surveys, and an interim usability questionnaire administered during early implementation, and they reflect the multifaceted impact of delegating alarm threshold-setting authority to nursing staff.

First, structured observations revealed a modest improvement in alarm response rates in the PICU, rising from 65 to 69%, alongside a shift toward more efficient and less disruptive response behaviors, such as visual checks and remote actions. In contrast, response rates in the GICU remained stable, though similar behavioral patterns were observed. Notably, these shifts were accompanied by slightly longer response times, likely reflecting more deliberate but less intrusive response strategies.

These findings may reflect a meaningful shift in the mode rather than the speed of alarm response, indicating a growing reliance on remote or visual assessment strategies rather than immediate bedside entry. Given that the intervention was designed to enhance the *quality* and *appropriateness* of responses rather than to reduce response time per se, the modest increase in average response duration is not necessarily indicative of diminished responsiveness. Rather, it may suggest a more deliberate and prioritized approach to alarm management aligned with clinical relevance.

Second, pre- and post-intervention survey data showed mixed trends: while some alarm fatigue indicators persisted or increased, these may reflect heightened staff awareness and critical reflection on alarm management practices following the training. Finally, responses to the interim implementation and usability survey indicated high perceived clarity and applicability of the training module, increased confidence among nurses in independently setting alarm thresholds, and strong support from physicians. Taken together, these results suggest that the intervention was feasible, acceptable, and partially effective in improving alarm engagement and promoting shared responsibility, though outcomes varied between units.

Improving alarm responsiveness is a central concern in ICU safety, as reduced sensitivity to alarms has been linked to missed critical events. Our focus on behavioral response outcomes reflects the urgency of this issue in high-acuity environments. The intervention’s outcomes differed markedly between units. The PICU demonstrated more significant improvements, with notable reductions in alarm fatigue and higher performance metrics, staff satisfaction, and training comprehension. In contrast, the GICU showed minimal changes, likely due to pre-existing informal practices of nurse-managed alarm thresholds. These differential responses underscore the critical importance of tailoring interventions to unit-specific organizational dynamics, as suggested by Weiner’s research on organizational change readiness [34].

A paradoxical finding was the simultaneous improvement in alarm attentiveness and increased awareness of alarm fatigue. This aligns with Casey et al.‘s observation that awareness interventions may initially amplify perceived issues while simultaneously reducing actual fatigue levels [35]. The complexity of addressing alarm fatigue is evident, highlighting the need for sustained, context-sensitive implementation strategies.

The study’s findings are consistent with broader literature highlighting alarm fatigue as a significant patient safety concern. With up to 99% of hospital alarms being nonactionable [36, 37], interventions that enhance alarm management are crucial for maintaining healthcare provider responsiveness and patient safety.

Our findings align with global trends toward delegating specific responsibilities from physicians to nurses. Examples include Bi et al.‘s study in China [19] and subsequent study from Ghana and Lebanon [2, 38]. However, this study is groundbreaking in Israel as the first to formally evaluate the delegation of alarm threshold authority with institutional support. This is particularly significant in the traditionally hierarchical Israeli healthcare system, demonstrating that such initiatives can shift established roles and improve care delivery dynamics. These findings parallel international trends toward increased nursing autonomy in specialized care areas, including neonatal and cardiac ICUs, where similar interventions have shown promise in improving both staff satisfaction and patient outcomes.

An unexpected psychological dimension emerged: some nursing staff experienced decreased self-confidence despite demonstrating competence in threshold management. This mirrors Lu et al.‘s observations about authority delegation’s potential psychological impacts [39]. Conversely, physicians uniformly supported the initiative, recognizing enhanced nurse attentiveness and alarm management. Physicians, however, uniformly supported delegating responsibility to appropriately trained nursing staff, acknowledging enhanced nurse attentiveness and patience with alarms when responsible for threshold adjustments. This aligns with a comprehensive study which demonstrated that nursing staff empowerment leads to improved patient outcomes and staff satisfaction [40].

Although the overall rate of addressed alarms increased only modestly in the PICU and remained unchanged in the GICU, the nature of staff responses shifted toward more immediate, yet less intrusive behaviors (e.g., head-raising and remote silencing). These trends suggest an improvement in alarm prioritization and situational awareness rather than a binary change in responsiveness. The findings support the feasibility of transferring alarm-setting authorities to nurses, while highlighting the complexity of measuring behavioral change in dynamic ICU settings. The study’s strengths include its use of objective observational data, a pre-post intervention design, and the simultaneous evaluation of two distinct ICU units, allowing for direct comparisons. These methodological choices provide a robust foundation for understanding the impact of authority delegation on alarm fatigue, staff autonomy, and overall care quality.

While offering valuable insights, this study acknowledges several methodological and contextual limitations. The single-center design and relatively small sample size limit the generalizability of the findings. Observational data were collected only from visible, occupied beds (1–5 per session), and clinical attributes of the patients involved were not recorded. As a result, we could not assess whether alarm responses varied according to patient acuity or risk level.

We also did not document the total unit census during each observation session, preventing accurate calculation of patient-to-nurse ratios across the unit. While the number of nurses on duty was recorded, workload variability may have influenced staff responsiveness. Additionally, survey responses were anonymous and could not be matched over time, limiting the ability to assess within-subject change. Post-intervention response rates were lower than at baseline, introducing potential self-selection bias.

Finally, the study was conducted during a period of healthcare system strain, including the COVID-19 pandemic and regional conflict, which may have affected staff engagement and implementation. The study’s setting within two ICUs of a single Israeli public hospital also reflects a unique organizational culture, potentially limiting external applicability. An additional methodological limitation relates to the survey tool itself. Although the survey instrument was developed based on existing validated questionnaires and underwent expert review and pilot testing, it has not yet been fully validated psychometrically. As such, the reliability and construct validity of the tool require further examination in future research.

The study’s implementation during a period of substantial healthcare system strain, specifically the COVID-19 pandemic and the 2023 war in Israel, may have further impacted staff engagement and protocol adaptation. The study’s implementation during a period of substantial healthcare system strain, specifically COVID-19 pandemic and the 2023 war in Israel, potentially impacted staff engagement and protocol adaptation. The research’s generalizability is further complicated by the unique characteristics of the healthcare environment. Conducted within two distinct ICU settings in a single Israeli public hospital, the study reflects a specific organizational culture with potentially unique power dynamics that may not directly translate across different institutional and national contexts.

Future policy development should focus on national guidelines that support nursing autonomy in alarm management, while integrating advanced technologies, such as AI-based alarm management systems, to further reduce alarm fatigue and enhance efficiency. Longitudinal studies and multi-site investigations examining the long-term impacts of authority delegation on patient outcomes and staff satisfaction are essential to validate and expand upon these findings.

Moreover, the study underscores the importance of structured training in empowering nurses to manage alarm settings effectively. While most nurses reported increased confidence and attentiveness to alarms post-intervention, some experienced initial uncertainty, emphasizing the need for ongoing support and reinforcement. Importantly, physicians acknowledged the benefits of the intervention, though they continued to prefer collaborative decision-making on alarm settings.

Given the variability in alarm management practices across Israeli hospitals and the absence of national guidelines, these findings support the need for standardized policies that formally recognize and regulate nurses’ authority in this domain. Future research should explore long-term impacts on patient outcomes, staff well-being, and alarm fatigue reduction, as well as the potential integration of AI-driven alarm management tools to further optimize ICU environments.

## Conclusions

This study demonstrated the feasibility and acceptability of delegating alarm threshold-setting authority to ICU nursing staff within a structured training and policy framework. The intervention led to improved behavioral engagement with alarms and was positively perceived by both nurses and physicians. These findings contribute to the literature on nurse empowerment and alarm management, offering a scalable model for shared clinical responsibility in high-acuity settings.

## Supplementary Information

Below is the link to the electronic supplementary material.

Supplementary Material 1

Supplementary Material 2

Supplementary Material 3

## Data Availability

The datasets generated and/or analyzed during the current study are available from the corresponding author on reasonable request.

## Abbreviations

AACN: American Association of Critical Care Nurses
AI: Artificial Intelligence
FDA: Food and Drug Administration
GICU: General Intensive Care Unit
ICU: Intensive Care Unit
JCI: Joint Commission International
NIHP: National Institute for Health Policy
NICU: Neonatal Intensive Care Unit
PICU: Pediatric Intensive Care Unit
