# Supplementary material for: Alarm fatigue mitigation through nurse empowerment: a pre-post intervention study in two intensive care units
Source: BMC Nurs. 2025 Aug 5;24:1022. doi: 10.1186/s12912-025-03613-9 (PMC12323265; doi:10.1186/s12912-025-03613-9)
Supplement: Supplementary file 1 — Supplementary Material 1 [file 12912_2025_3613_MOESM1_ESM.pdf]

Implementation and Training Module Questionnaire

(Two Weeks Post-Intervention)

Date of survey completion: ______________________________

Year of your birth: ______________________________

Gender: ______________________________

Unit: General ICU / Pediatric ICU

Section A: Questions for Nurses

Scale:

- Not at all relevant
- Strongly disagree
- Disagree
- Agree
- Strongly agree

Statement
I have completed the training module on alarm threshold setting.
The training module was clear and understandable to me.
The module was easy to complete.
The module conveyed the principles of alarm threshold setting well.
Over the past week, I have independently set alarm thresholds.
I feel confident setting alarm thresholds for my patients on my own.
I am satisfied with the ability to set alarm thresholds independently.
I still consult with the physician about setting alarm thresholds.
Despite the training, I still find setting alarm thresholds to be a complex task.
I feel more attentive to alarms and beeps now that I set alarm thresholds myself.
I feel more patient toward alarms and beeps since I have been setting the thresholds.
I feel that I trust alarms and beeps more since I have been setting the thresholds.

Section B: Questions for Physicians

Scale:

- Strongly disagree
- Disagree
- Agree
- Strongly agree

Statement
I trust the nurses in our unit to appropriately set alarm thresholds for patients now that they have received proper training.
I also trusted the nurses to do so before the training module and before the new policy was officially introduced.
I still prefer that nurses consult with me before changing alarm thresholds.
I feel that the nurses are now more attentive and patient toward alarms and beeps since they began setting alarm thresholds.
