# Supplementary material for: Alarm fatigue mitigation through nurse empowerment: a pre-post intervention study in two intensive care units
Source: BMC Nurs. 2025 Aug 5;24:1022. doi: 10.1186/s12912-025-03613-9 (PMC12323265; doi:10.1186/s12912-025-03613-9)
Supplement: Supplementary file 2 — Supplementary Material 2 [file 12912_2025_3613_MOESM2_ESM.pdf]

Alarm Fatigue Questionnaire – English Version

Scale: Strongly agree > Agree> Disagree> Strongly disagree > Not relevant

Section A: General Exposure and Attitudes Toward Alarms

Instructions: Please indicate your level of agreement with each statement.

No.	Statement
1	I frequently experience repetitive alarms during my work.
2	Repetitive alarms interfere with patient care.
3	Repetitive alarms reduce my trust in them.
4	Repetitive alarms lead staff to mute or disable them.
5	Staff are sensitive to alarms and respond quickly.
6	Background noise makes it harder for me to detect alarms.
7	It is difficult to set alarm thresholds as desired.

Section B: Personal Reactions to Alarm Exposure

The following questions refer to your personal reactions to alarm exposure.

No.	Statement
8	When alarms repeat continuously, I lose my patience.
9	When alarms are frequent, I become indifferent to them.
10	I approach the patient's bedside immediately upon hearing an alarm.
11	Multiple simultaneous alarms confuse me when I need to make decisions.
12	Alarm sounds prevent me from optimally performing my professional role.
13	Alarm sounds cause me to feel anxious.
14	I am confident that the alarm thresholds are correctly set.
15	I sometimes delay responding to an alarm, hoping it will stop on its own.
16	I respond only to prolonged red alarms.
17	I respond immediately to ventilator alarms.
18	I respond immediately to monitor alarms.

19	At times, the workload in the unit prevents me from responding quickly.
20	I try to differentiate between informational (yellow) and warning (red) alarms.
21	Over time, I feel less sensitive to alarms.
22	I believe most of the noise in the unit originates from monitoring devices.
23	Sometimes I do not hear the alarm at all.
24	A high alarm volume increases the likelihood that I will respond to it.

Section C: Alarm Policy and Practices

No.	Statement
25	My department makes effective use of alarm management protocols and policies.
26	At the start of my shift, I tend to set wider alarm thresholds than recommended.
27	I believe that nurses (in addition to physicians) should be authorized to set alarm thresholds.

Section D: Demographics

Year of birth: ________________

Gender: ______________________

Academic education: ______________________

Unit: General ICU / Pediatric ICU

Role: Nurse / Physician / Other: ___________
