# Supplementary material for: Alarm fatigue mitigation through nurse empowerment: a pre-post intervention study in two intensive care units
Source: BMC Nurs. 2025 Aug 5;24:1022. doi: 10.1186/s12912-025-03613-9 (PMC12323265; doi:10.1186/s12912-025-03613-9)
Supplement: Supplementary file 3 — Supplementary Material 3 [file 12912_2025_3613_MOESM3_ESM.pdf]

# Standards for Reporting Implementation Studies: the StaRI checklist for completion

The StaRI standard should be referenced as: Pinnock H, Barwick M, Carpenter C, Eldridge S, Grandes G, Griffiths CJ, Rycroft-Malone J, Meissner P, Murray E, Patel A, Sheikh A, Taylor SJC for the StaRI Group. Standards for Reporting Implementation Studies (StaRI) statement. *BMJ* 2017;356:i6795

The detailed Explanation and Elaboration document, which provides the rationale and exemplar text for all these items is: Pinnock H, Barwick M, Carpenter C, Eldridge S, Grandes G, Griffiths C, Rycroft-Malone J, Meissner P, Murray E, Patel A, Sheikh A, Taylor S, for the StaRI group. Standards for Reporting Implementation Studies (StaRI). Explanation and Elaboration document. *BMJ Open* 2017 2017;7:e013318

| Checklist item | | Reported on page # | Implementation Strategy | Reported on page # | Intervention |
|---|---|---|---|---|---|
| | | | "Implementation strategy" refers to how the intervention was implemented | | "Intervention" refers to the healthcare or public health intervention that is being implemented. |
| **Title and abstract** | | | | | |
| Title | 1 | 1 | Identification as an implementation study, and description of the methodology in the title and/or keywords | | |
| Abstract | 2 | 2 | Identification as an implementation study, including a description of the implementation strategy to be tested, the evidence-based intervention being implemented, and defining the key implementation and health outcomes. | | |
| **Introduction** | | | | | |
| Introduction | 3 | 3-4 | Description of the problem, challenge or deficiency in healthcare or public health that the intervention being implemented aims to address. | | |
| Rationale | 4 | 4-5 | The scientific background and rationale for the implementation strategy (including any underpinning theory/framework/model, how it is expected to achieve its effects and any pilot work). | 4-5 | The scientific background and rationale for the intervention being implemented (including evidence about its effectiveness and how it is expected to achieve its effects). |
| Aims and objectives | 5 | 6 | The aims of the study, differentiating between implementation objectives and any intervention objectives. | | |
| **Methods: description** | | | | | |
| Design | 6 | 6 | The design and key features of the evaluation, (cross referencing to any appropriate methodology reporting standards) and any changes to study protocol, with reasons | | |
| Context | 7 | 6-7,25 | The context in which the intervention was implemented. (Consider social, economic, policy, healthcare, organisational barriers and facilitators that might influence implementation elsewhere). | | |

| | | | | | |
|---|---|---|---|---|---|
| Targeted 'sites' | 8 | 6-7 | The characteristics of the targeted 'site(s)' (e.g locations/personnel/resources etc.) for implementation and any eligibility criteria. | 6-7 | The population targeted by the intervention and any eligibility criteria. |
| Description | 9 | 7-8 | A description of the implementation strategy | 7-8 | A description of the intervention |
| Sub-groups | 10 | 6 | Any sub-groups recruited for additional research tasks, and/or nested studies are described | | |
| **Methods: evaluation** | | | | | |
| Outcomes | 11 | 9-12 | Defined pre-specified primary and other outcome(s) of the implementation strategy, and how they were assessed. Document any pre-determined targets | 9-12 | Defined pre-specified primary and other outcome(s) of the intervention (if assessed), and how they were assessed. Document any pre-determined targets |
| Process evaluation | 12 | 9-12 | Process evaluation objectives and outcomes related to the mechanism by which the strategy is expected to work | | |
| Economic evaluation | 13 | - | Methods for resource use, costs, economic outcomes and analysis for the implementation strategy | - | Methods for resource use, costs, economic outcomes and analysis for the intervention |
| Sample size | 14 | 12-13 | Rationale for sample sizes (including sample size calculations, budgetary constraints, practical considerations, data saturation, as appropriate) | | |
| Analysis | 15 | 13 | Methods of analysis (with reasons for that choice) | | |
| Sub-group analyses | 16 | - | Any a priori sub-group analyses (e.g. between different sites in a multicentre study, different clinical or demographic populations), and sub-groups recruited to specific nested research tasks | | |
| **Results** | | | | | |
| Characteristics | 17 | 14,16 | Proportion recruited and characteristics of the recipient population for the implementation strategy | 14,16 | Proportion recruited and characteristics (if appropriate) of the recipient population for the intervention |
| Outcomes | 18 | 21 | Primary and other outcome(s) of the implementation strategy | 14-21 | Primary and other outcome(s) of the Intervention (if assessed) |
| Process outcomes | 19 | 21 | Process data related to the implementation strategy mapped to the mechanism by which the strategy is expected to work | | |
| Economic evaluation | 20 | - | Resource use, costs, economic outcomes and analysis for the implementation strategy | - | Resource use, costs, economic outcomes and analysis for the intervention |
| Sub-group analyses | 21 | 14-21 | Representativeness and outcomes of subgroups including those recruited to specific research tasks | | |
| Fidelity/ adaptation | 22 | 21 | Fidelity to implementation strategy as planned and adaptation to suit context and preferences | 21 | Fidelity to delivering the core components of intervention (where measured) |

| | | | | | |
|---|---|---|---|---|---|
| Contextual changes | **23** | **25** | Contextual changes (if any) which may have affected outcomes | | |
| Harms | **24** | **-** | All important harms or unintended effects in each group | | |
| **Discussion** | | | | | |
| Structured discussion | **25** | **22-26** | Summary of findings, strengths and limitations, comparisons with other studies, conclusions and implications | | |
| Implications | **26** | **26-27** | Discussion of policy, practice and/or research implications of the implementation strategy (specifically including scalability) | **26-27** | Discussion of policy, practice and/or research implications of the intervention (specifically including sustainability) |
| **General** | | | | | |
| Statements | **27** | **28** | Include statement(s) on regulatory approvals (including, as appropriate, ethical approval, confidential use of routine data, governance approval), trial/study registration (availability of protocol), funding and conflicts of interest | | |
